# Study of Cement-Based Superhydrophobic Composite Coating: New Option for Water Drainage Pipeline Rehabilitation

**DOI:** 10.3390/ma13215004

**Published:** 2020-11-06

**Authors:** Tianyu Wang, Cong Zeng

**Affiliations:** Department of Engineering, China University of Geosciences, Wuhan 430074, China; cugwty@cug.edu.cn

**Keywords:** superhydrophobicity, SiO_2_ aerosol @ bisphenol A diglycidyl ether coating, water drainage pipe, cement-based materials

## Abstract

A great number of urban underground concrete water drainage systems in China are facing challenges of corrosion, blockage, and leakage. This could result in engineering accidents such as urban inland inundation, pipeline collapse, leakage, and blockage. The common contributing factors for pipeline leakage and blockage are the porous structures and the perishable surfaces of concrete pipes. To address these issues, we synthesized superhydrophobic coating materials such as SiO_2_ aerosol, bisphenol A diglycidyl ether (DGEBA), and N-β-aminoethyl-γ-aminopropyltrimethoxysilane (AEAPTS). Our superhydrophobic coating on cement-based surfaces presents good waterproof ability, mechanical stability, and self-cleaning properties. Test results show that the superhydrophobic coating exhibits higher water discharge capacity and survivability to corrosive underground water drainage pipeline environments. Hence, this SiO_2_ aerosol @ bisphenol A diglycidyl ether coating possesses enormous potential in surface modification of pipeline rehabilitation materials.

## 1. Introduction

Many infrastructures in China are in a severely poor condition, making rehabilitation work desperately needed. This situation is worse in the underground structures, which are facing harsh working environments such as water infiltration, corrosive soil, and disturbing loads. The deterioration of cement-based materials would be accelerated when water and other chemicals penetrate the pores of the matrix [1]. Most of the water drainage pipes in China have problems with leakage, corrosion, and structural instability, which could result in wasting resources, environmental pollution, and a great loss of social-economic benefit [2,3]. To address these issues, many trenchless methods have been widely used such as cured-in-place pipes, sealers, and coatings [4]. The coating method is getting more and more attention in the field because of its effective construction, economic friendliness, and wide applicability [5]. Among those existing coating materials, fiber-reinforced cement mortar and epoxy resin material can together help to provide structural support, controlling the water quality while showing good compatibility with original materials [6,7]. However, corrosion resistance has always been a major problem in water drainage pipeline rehabilitation as the cement-based repairing liner is prone to corrosion again. What is more, the cement-based coating will reduce the inner diameter of the pipe, leading to flow loss [4]. Therefore, the surface corrosion resistance and discharge capacity of the repairing material are the key elements of the rehabilitation work.

The SiO_2_ aerogel was widely used as the modifier in cement-based materials [8], and previous studies demonstrated the influence of SiO_2_ aerogel on durability, density, mechanical properties, thermal conductivity, and other properties of the concrete mixture [9,10]. However, it seems to only delay the corrosion to a certain extent rather than insulate it. The concept of superhydrophobic hybridization incorporates biomimicry (lotus effect) with a water contact angle (WCA) greater than 150° and a sliding angle (SA) less than 10°, mimicking lotus leaves and water striders, which have gained attention from people in the superhydrophobic field [11,12,13,14]. Superhydrophobic coatings enable engineers to improve the durability, self-cleaning, and discharge capacity of cement-based materials, thus effectively reducing the damage caused by the all-pervasive corrosive fluid and dirt in sewage environments [15,16,17,18]. Among those techniques such as the hydrothermal technique [19], etching [20], assembling [21], phase separation [22], chemical vapor deposition [23], and electrochemical [24], the coating is the simplest and most economical option, making it an exemplary candidate for engineering practice [25]. A variety of experiments were performed to create the superhydrophobic surface and the characteristics such as durability, frost-resistance, and abrasive resistance generated enormous research interest. For example, Wong et al. designed a waterproof concrete by using the superhydrophobic paper sludge ash [26]. Husni et al. extracted the silica nanoparticles from rice husk ash and created a superhydrophobic coating that improves the anti-permeability of the concrete [18]. She et al. fashioned a mechanically durable superhydrophobic concrete surface by a direct spraying method [27]. Arabzadeh et al. coated the concrete substrates by using the layer-by-layer deposition technique [28]. Young et al. found that the friction responses and roughness of monodispersed coatings were lower than those of hierarchically structured coatings [29].

However, not enough research has been laid on the real-world applications of superhydrophobic cement-based materials under complex working conditions. For example, there is very limited working space in underground water drainage pipeline rehabilitation work, which means that the superhydrophobic coating should be combined with the trenchless method [2]. The resistance of the coating to corrosive flow and the impact the coating might have to the cement-based rehabilitation material should also be considered [3,4].

In our current study, we utilized a nontoxic method to fashion a fiber-reinforced cement mortar surface modified by a SiO_2_ aerosol @ bisphenol A diglycidyl ether hierarchically structured coating. This superhydrophobic coating consisted of pipeline structure rehabilitation materials possessing good mechanical, antifouling, discharge capacity, and corrosion resistance while the superhydrophobic modification had few impacts on the mechanical performance of the rehabilitation materials. This simple, effective, and economical method can be operated in a beaker, and the anti-corrosion property and mechanical durability of the coating were demonstrated when exposed to special conditions mimicking those of underground water drainage pipelines. Our findings provide significant insight into the application of superhydrophobic materials in trenchless rehabilitation work.

## 2. Materials and Methods

### 2.1. Cementitious Materials

In the study, the 40 × 12 Kuralon K-II PVA fibers (diameter: 40 μm, length: 12 mm) from Kuraray (Tokyo, Japan) were used. This kind of fiber has a 1.6 GPa tensile strength and Young’s modulus of more than 38 GPa. Another component of the pipeline repairing fiber-reinforced cement mortar mixtures was Portland cement from Longlin Cement (Longyan, China) conforming to ASTM C150 [30]. Fly ash was from Newreach Materials (Wuhan, China) and the chemicals were SiO_2_ (58%), Al_2_O_3_ (30%), Fe_2_O_3_ (4.3%), CaO (1.5%), MgO (2.8%), and Na_2_O (3.2%). Silica sand (Hengwang Environmental Protection, Zhengzhou, China) with a SiO_2_ content more than 99.3% and an average particle size of 0.5 mm was used. The polycarboxylate superplasticizer was from SUNBO (Suzhou, China) with a water-reducing effect of 30%.

### 2.2. Coating Materials

The silane coupling agent (AEAPTS) was bought from Aladdin (Shanghai, China); bisphenol A, epichlorohydrin, and the curing agent were provided by RESIPLAST PROTECT (Antwerp, Belgium) and XIAMEN ANYUE (Xiamen, China); 38% hydrochloric acid (HCl), absolute ethyl alcohol, ≥28% ammonium hydroxide, and 97% sheet sodium hydroxide were bought from Xilong Scientific Co., Ltd.(Guangdong, China); SiO_2_ aerogel materials were bought from Pomeloking Eco-Technologies (Qingyuan) Co., Ltd. (Qingyuan, China).

#### 2.2.1. The Synthesis Method of SiO_2_ Aerogel Superhydrophobic Powder

One of the important influences on the superhydrophobicity of the coating is the mass ratio of SiO_2_ aerogel and AEAPTS. Hence, five different solutions were designed, as shown in Table 1, to evaluate this influence. First, ethyl alcohol (5 mL) and SiO_2_ aerogel were added into a beaker at the same time and stirred for 1 min. Then, AEAPTS was put into the solution with 10 min of stirring. A certain amount of ammonium hydroxide was added drop-by-drop into the beaker to promote the hydrolysis reaction of the AEAPTS and make the solution gel. It was designed to keep stirring for more than 6 h to prepare the colloidal solution successfully.

The colloidal solution was dripped into glass substrates to be evenly spread. Then, the samples were put into an oven and dried at 60 °C for 6 h until the ethanol completely evaporated, and after that, the residue was ground into powders and collected.

#### 2.2.2. Fabrication of SiO_2_ @ DGEBA Superhydrophobic Coatings

The curing agent and DGEBA (1:2) were put into a certain amount of ethanol together to prepare a 3% DGEBA solution with 5 min of stirring. After that, the solution was sprayed evenly onto the cement specimens’ surfaces as the adhesive. Then, the superhydrophobic powder (0.3 g per time) was laid evenly om the adhesive layer 3–5 times after 30 min, and the excess blown off by a blower. After the composite material was bone dry, a superhydrophobic coating was formed on the surface of the matrix.

### 2.3. Cement Samples

The cement specimens were produced at a water–cement ratio of 0.30 and a sand–cement ratio of 0.7. The 15% cement quantity was replaced by fly ash, and the dosage of polycarboxylate superplasticizer was determined as 0.8% of the quality of cementing material. The specimens were cast in 40 mm × 40 mm × 40 mm cube molds. The volume ratio of PVA fibers was 1.75%. These kinds of cement-based materials have been used to repair damaged water drainage pipelines in America, China, and many other countries [31]. To endow the cementitious composite with a better performance in rehabilitation work, specimens were divided into four groups (Table 2) with the surface special handing-treatment.

**Figure 1:**
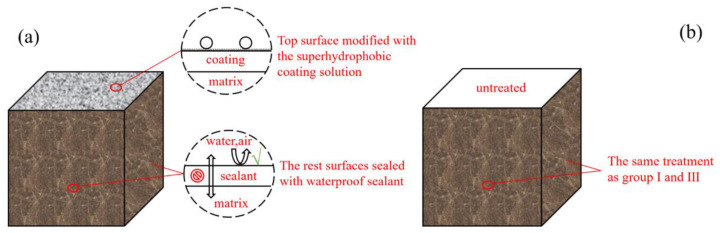
(**a**) Preparation of the treatment of the specimens in group I and III; (**b**) the top surfaces of specimens in group III and IV were untreated.

Upon compaction, the samples were cured at a room temperature of 20 ± 5 °C and relative humidity of more than 95%. Then, the specimens were demolded 24 h later as the modification was completed. The waterproof sealant was used to ensure that the top surfaces of the cube specimens were the only area in contact with the curing environment and at which moisture ion transport occurs (see Figure 1). Several parameters were tested to observe the effect of the coating on the specimens under different curing conditions.

### 2.4. Characterization Methods

A Tecnai G2 TF20 S-TWIN (Park Systems, Suwon, South Korea) was operated at 300 kV to carry out the transmission electron microscopy (TEM) testing. The specimens were prepared and Au-sputtered, then measured by a JSM-6701F (JEOL Ltd., Tokyo, Japan) to obtain the FESEM (field emission scanning electron microscope) images. A Thermo Scientific ESCALAB 250Xi (Thermo Scientific, Waltham, MA, USA) was used for the XPS (X-ray photoelectron spectroscopy) measurement while the excitation source was the Al Ka line. A Thermo Scientific Nicolet iS10 (Thermo Scientific, Waltham, MA, USA) was used to record the FTIR (Fourier-transform infrared spectra) spectroscopy. A JSM-5601LV SEM (scanning electron microscope, JEOL Ltd., Tokyo, Japan) was operated at 5 keV to obtain the SEM images of platinum-plated samples. A JC2000D (Shanghai Zhongchen Technology Apparatus Co., Ltd., Shanghai, China) was used to measure the water contact angles (WCA) of different types of samples at ambient temperature. Optical photographs were taken by a Nikon D7200 (Tokyo, Japan). These experimental works were done in Hubei Key Laboratory of Polymer Materials, Hubei University (Wuhan, China).

In accordance with ASTM C109, the 40 mm × 40 mm × 40 mm cubes were tested at a 0.9 kN/s loading rate at 7, 28, 60, 90 and 120 days to obtain the compressive strength. The water absorption value and quality loss were also measured as important indicators in accordance with ASTM C642 [32].

## 3. Results

### 3.1. Structure and Composition of the Adhesive

Epoxy resin is well-known for its applications in coating, adhesives, aerospace, and the electronics industry because of its outstanding physicochemical properties [32]. DGEBA provided by RESIPLAS PROTECT (Antwerp, Belgium) and XIAMEN ANYUE (Xiamen, China) was used in this study. It was obtained through condensation of bisphenol A and epichlorohydrin under alkaline conditions. The DGBPA used in this study was opaque white pasty fluid, which turned to a whitish-gray solid after curing. The epoxy equivalent weight (EEW) and the epoxy value of the DGBPA were 210–280 g/mol and 0.357–0.476 mol/100 g, respectively, and it showed a melting point of 205–272 °C and viscosity of 5000–10,000 mPa·s.

**Figure 2:**
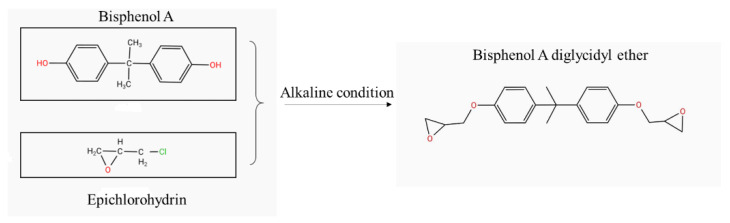
The reaction mechanism of bisphenol A diglycidyl ether (DGEBA).

The reaction mechanism is shown in Figure 2. The special molecular structure of DGEBA gave it better mechanical property, corrosion resistance, and adhesion performances. Those advantages made DGEBA suitable for application in underground infrastructure rehabilitation work. The pilot experiment had been carried out to evaluate the performance of DGEBA as the adhesive.

**Figure 3:**
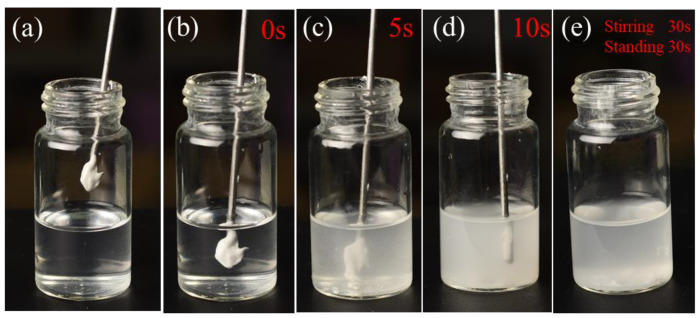
The first 30 s when DGEBA was dispersed in ethyl alcohol (**a**–**e**).

**Figure 4:**
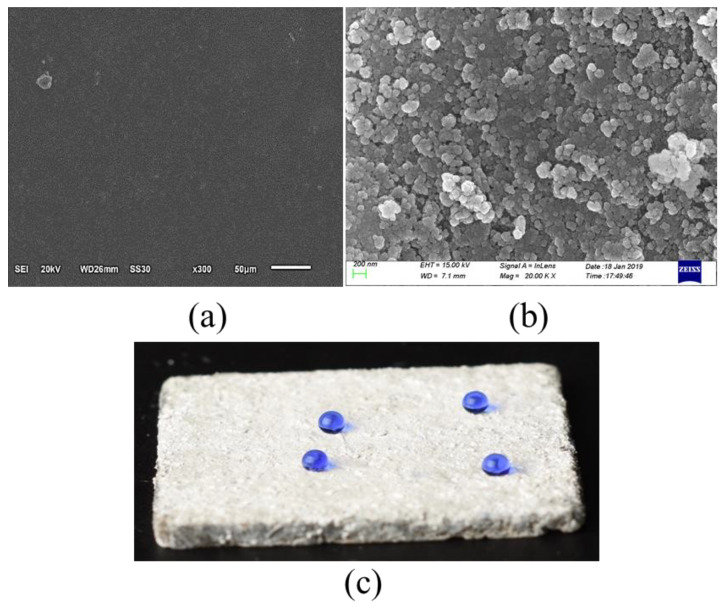
(**a**) DGEBA layer on cement sample’s surface; (**b**) FESEM images of superhydrophobic particles on DGEBA; (**c**) SiO_2_ aerosol @ DGEBA coating.

The DGEBA could disperse uniformly in ethyl alcohol with simple stirring at room temperature, as shown in Figure 3. After drying, the DGEBA solution adhered to the matrix smoothly (Figure 4a). Figure 4b,c show that the superhydrophobic particles were observed on the surface of the DGEBA coating.

Results show that the DGEBA exhibited a significant performance in dispersing, film formation, and adhesion between the superhydrophobic powder and cement samples. Therefore, the DGEBA could be an ideal adhesive in the following surface modification experiment.

### 3.2. Superhydrophobicity of the Modified SiO_2_ Aerogel Powder

The hydrolysis reaction of the AEAPTS promoted the formation of Si-O-Si-(NH_2_(CH_2_)_2_NH(CH_2_)_3_) linkages and showed that parts of hydrophilic groups (Si-OH) would be transformed into hydrophobic groups.

**Figure 5:**
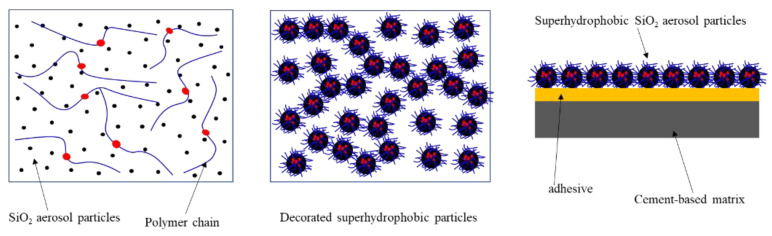
A schematic diagram for the preparation of compound SiO_2_ @ DGEBA superhydrophobic coating.

AEAPTS hydrolytic reaction generated Si-O-Si-(NH_2_(CH_2_)_2_NH(CH_2_)_3_) on the surface of the particles, which brought superhydrophobicity to the powder. Then, the papillary structure was formed when the surface of the adhesive was evenly covered by these superhydrophobic particles, increasing the roughness of the coating, as shown in Figure 5. The superhydrophobicity is attributed to the nano and microscale hierarchical structure based on the wetting theory developed by Cassie and Baxter [33].

**Figure 6:**
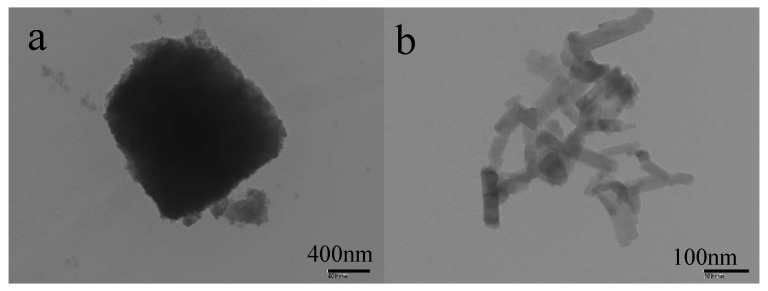
(**a**) Superhydrophobic particles with a rough surface; (**b**) The chain-type superhydrophobic particles.

TEM images (Figure 6) showed that the coating consisted of countless superhydrophobic particles, and the rough surface and the low surface energy also contributed to the superhydrophobic property.

**Figure 7:**
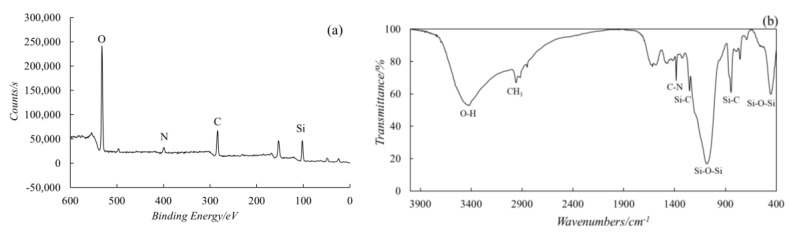
(**a**) XPS survey of the superhydrophobic particles; (**b**) FTIR image of the superhydrophobic particles.

The SiO_2_ aerogel superhydrophobic powder was characterized by XPS survey and FTIR analysis. The XPS image (Figure 7a) showed a peak of nitrogen around 400 eV, demonstrating that the AEAPTS had brought amino groups onto the particles. The carbon peak around 285 eV (Figure 7a) suggested that the hydrophobic alkyl was grafted on the surface of SiO_2_ particles after the hydrolysis of AEAPTS. An antisymmetric stretching vibration peak of O-H was observed at 3442 cm^−1^ according to the FTIR spectrum (Figure 7b). The Si-O-Si skeleton structure of SiO_2_ aerosol was confirmed by the transmittance peaks around 1084 and 455.1 cm^−1^. The appearance of CH_3_ and C-N’s peaks at 2958.4 and 1384.6/1322.9 cm^−1^, respectively, indicated that the hydrophilic hydroxyl had been replaced by the hydrophobic grouping, which was consistent with the results of XPS survey. This was also substantiated by the peak of Si-C at 1253 and 844 cm^−1^. The XPS survey and FTIR spectrum together showed that the surface of the SiO_2_ aerosol was covered by superhydrophobic structures that are necessary for the formation of a stable solution and superhydrophobic compound particles.

**Figure 8:**
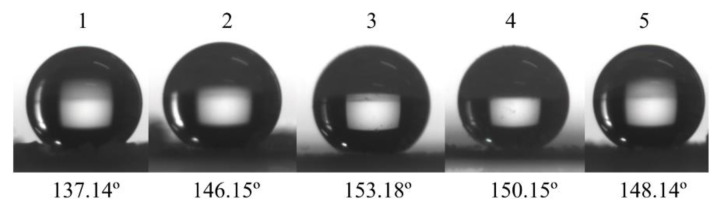
Water contact angle (WCA) images of five different groups.

Cement samples were modified by using five groups of superhydrophobic powder designed according to Table 1 and the DGEBA. The WCAs of all five samples are shown in Figure 8. Group 3 exhibited the best superhydrophobicity among all groups, with the WCA reaching 153°. In addition, WCA images (Figure 8) demonstrate that the appropriate mass ratio of the SiO_2_ aerosol and AEAPTS creates better superhydrophobicity, while too much AEAPTS might impair it.

**Figure 9:**
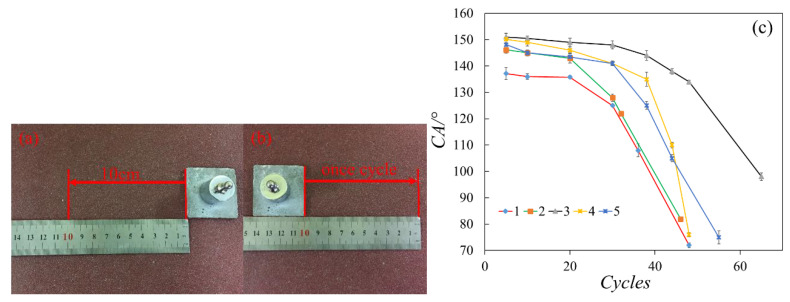
(**a**,**b**) Schematic image of the mechanical strength and binding force testing procedures; (**c**) WCA changes after friction.

In the quantitative testing of the different coatings’ binding force and mechanical strength, sandpapers (800 Cw) and a quantitative weight (50 g) were used in the tests. Figure 9a,b showed that the modified cement sample (40 mm × 40 mm) was put onto the sandpaper, and the sandpaper would directly contact the coated surface. The 50 g weight was added above the uncoated side and the specimen was pushed and slid slowly and evenly toward the ruler direction to 10 cm. Then, the coatings’ binding force and mechanical strength were tested by repeating the whole process several times as designed.

Figure 9c showed that the WCA value did not decrease significantly after 20 cycles. Besides, no obvious damage on the coating surface was observed with only a small amount of powder worn off from the specimens. While Group 3 and 4 hold a WCA higher than 148°, the WCA of Group 1 decreased rapidly after 24 cycles, and the WCA in Group 2 reduced after 32 cycles. The WCA in Groups 4 and 5 sustained more than 38 cycles while Group 3 maintained a high WCA even after 50 moving cycles.

The results of the quantitative testing (Figure 9) show that the coatings’ WCA was not affected by the amount of DGEBA [34], while the property of superhydrophobic powder had a significant impact on the superhydrophobicity of the coating when using the same adhesive. According to the test results, Group 3 held the best mechanical stability and superhydrophobicity, and it was chosen for the following test.

**Figure 10:**
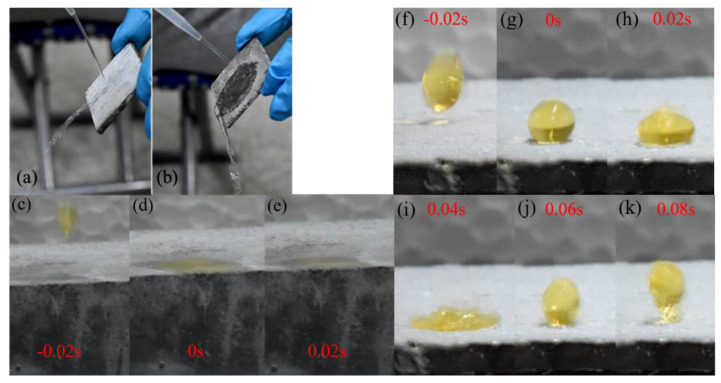
(**a**) Water column bouncing off the coated surface; (**b**) water column wetting the uncoated surface and spreading immediately. (**c**–**e**) Time-lapse photographs of water droplets spreading on the normal cement samples. (**f**–**k**) Time-lapse photographs of water droplets bouncing on the treated cement sample.

The previous study demonstrated that the water column bounces but does not wet or spread on the water-repellent surface [35], as shown in Figure 10a,b. In Figure 10c–k, we compared the water-affecting patterns between untreated and treated cement samples. Figure 10c–e illustrated that the water dropping process on the untreated cement samples surface readily wetted at the moment water droplets contacted the sample surface. The water droplet bounced off the treated surface without wetting, indicating the superhydrophobicity of the surface. The sizes of the droplets varied with random impact velocities.

### 3.3. Mechanical Performance and Quality Loss

**Figure 11:**
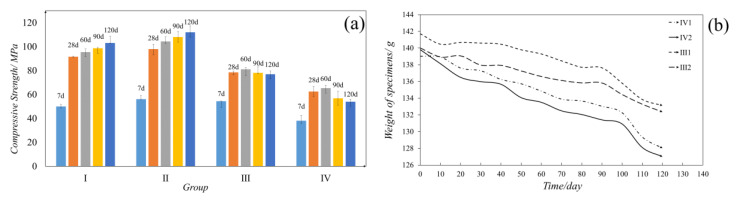
(**a**) Testing results of the cement samples’ compressive strength. (**b**) Weight changing of the cement samples.

The coating material functions as a protective layer that enables the cement-based rehabilitation material to resist the negative effects of the corrosive environment. Samples were prepared and tested according to the design in Table 2. The 28d compressive strengths of the group I–IV cement samples were 91.6, 98.0, 81.6, and 62.4 MPa, respectively (Figure 11a). The average compressive strength of group III was 30.6% higher than that of group IV, while the average of group I was about 92% of that of group II.

As the change in the samples’ quality was negligible in a standard environment, we focused on the differences in mass change of group III and IV (Figure 11b). Our results show that the modified samples held a better performance when curing under a corrosive environment as the quality loss was 71.4% (30d), 52.4% (90d), and 41.8% (120d) lower than those of the standard samples, respectively.

The results of mechanical performance and quality loss suggest that the superhydrophobic coating provides good protection by enhancing the mechanical strength of the cement-based materials. Moreover, the modification had minimal impact on the cement samples in the standard curing environment.

### 3.4. Durability and Other Characteristics of SiO_2_ Aerosol @ DGEBA Coating

The durability of the repairing materials has a critical influence on the long-term integrity of the infrastructures, especially with variations in temperature, humidity, and corrosive environmental factors [36,37]. Our test result shows that the DGEBA exhibits good performance in coating and adhesiveness, and it is also regarded as a durable material in practical applications.

**Figure 12:**
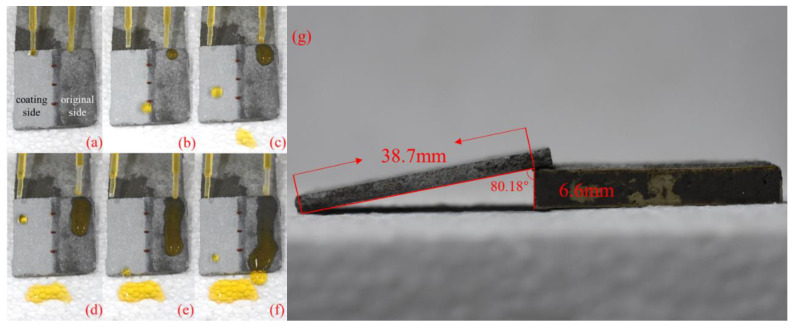
(**a**–**f**) The superhydrophobic surface holds a better drainage capacity compared to the untreated surface, (**g**) The side view of the half-coating specimen with a title angle of 9.82°.

Figure 12 shows the water droplets on a half-coating cement surface with a tilted angle of 9.82°. The water flowed faster on the superhydrophobic surface than the cement surface with the help of gravity. It can be seen from Figure 12 that the water droplets fell from the coating part quickly while the water stayed on the uncoated face. All the water droplets were trapped on the surface of the uncoated part while many droplets had passed through the coated part. This property of the superhydrophobic coating could significantly improve the drainage capacity of the urban drainage system, especially for those gravity pipelines [2,3].

**Figure 13:**
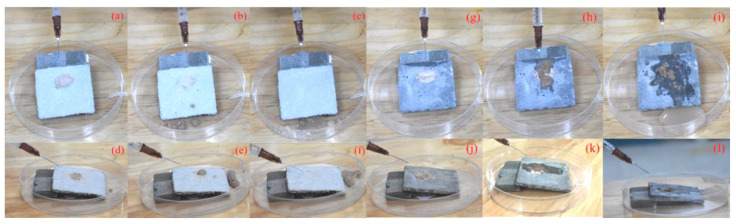
(**a**–**c**) The self-cleaning process on the SiO_2_ aerosol @ DGEBA, (**d**–**f**): Side view of the self-cleaning process on the SiO_2_ aerosol @ DGEBA, (**g**–**i**): The unsuccessful process of self-cleaning on the untreated cement surface, (**j**–**l**): Side view of the unsuccessful process of self-cleaning on the untreated cement surface.

The process of self-cleaning is shown in Figure 13. It can be seen in Figure 13a–l that the coating surface was full of clouds of dust. Then, an injection syringe was used to apply water dropwise onto the surface. The uncoated surface was wetted easily and remained dirty after a 5 min washing period (Figure 13g–l). As for the modified surface, the water drops could easily remove the dust from the specimen without wetting the surface (Figure 13a,b,d,e). Figure 13c,f show that all the dust was eventually washed out with much less cleaning water, and the surface remained clean after the whole process. The surface was not damaged and stayed dry during the experimental process, which also indicated the good durability of the coating.

**Figure 14:**
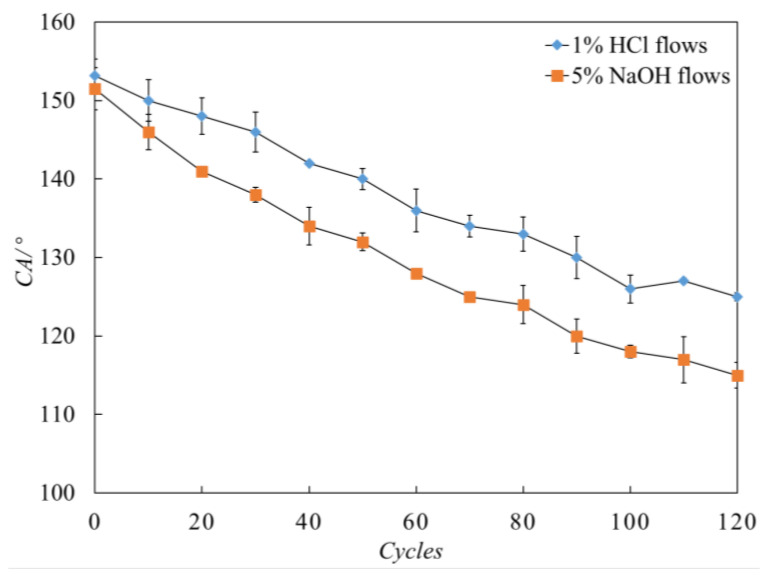
CA changes after 120 cycles.

To test the corrosion resistance capacity of the coating under an acid/alkaline sewer environment, the corrosive liquid was repeatedly applied onto the surface for 5 s each time. The specimens were found to maintain their self-cleaning capacity when the liquid changed from water to acid and lye (see Figure 14). Very small decrease of the contact angle (CA) value was observed after the first 10 cycles, and the surface sustained a high CA after 120 cycles (Figure 14).

## 4. Discussion

Our current study successfully prepared a robust SiO_2_ aerosol @ DGEBA coating based on the previous research [1,23,24,25,26]. The prepared coating presented excellent waterproof ability with a WCA greater than 150° and a roll position less than 10°. Our straightforward superhydrophobic coating preparation process did not rely on a special mold or mesh, and it also avoided spraying silane coupling agent in large quantities, which meets the requirements of mass production and applications in underground engineering environments [14,38,39]. In this paper, the superhydrophobic coating was designed closely combining with trenchless pipeline rehabilitation work. DGEBA was used as the ideal superhydrophobic coating adhesive [40]. The coating showed good mechanical stability, self-cleaning, water discharge capacity, and anti-corrosive properties, which meet the complex underground water drainage pipeline environment [2]. In the corrosive environment, the 28d compressive strength of samples under the protection of the coating was about 30.6% higher than those of the untreated samples, and the 28d quality loss of the modified group was 71.4% lower than that of the untreated group.

Results demonstrated that this SiO_2_ aerosol @ DGEBA coating holds great application prospects in underground water drainage pipeline rehabilitation work. This kind of interdisciplinary research combines the superhydrophobic materials, cement-based materials, and engineering practice together, which may further promote the real-world applications of the superhydrophobic materials. Besides, further research on the durability of the coating under long-term water erosion and field tests in the real-world environment of a water drainage pipeline are needed in the future.

## Figures and Tables

**Table 1:** Experimental design for superhydrophobic colloidal solutions.

Specimen ID	AEAPTS/g	SiO_2_ Aerogel/g	Ethyl Alcohol/mL
1	0.1	0.5	5
2	0.2	0.5	5
3	0.3	0.5	5
4	0.4	0.5	5
5	0.5	0.5	5

**Table 2:** Cement specimens design for durability testing.

Specimens ID	Top Surface	The Rest Ones	Curing Environment/pH
I	modified	Sealed	deionized water/approximately 7
II	untreated	Sealed	deionized water/approximately 7
III	modified	Sealed	sanitary sewage/3–5
IV	untreated	Sealed	sanitary sewage/3–5
